# Round the Hospitals

**Published:** 1917-09-01

**Authors:** 


### ROUND THE HOSPITALS.

The new Imperial Orders—*i.e.* the "Order of the British Empire" and the "Order of Companions of Honour"—have now been brought to birth, and a list of the first recipients of them has been published in the *Gazette*. Further, there has been created the "Medal of the Order of the British Empire" for services of special merit—acts displaying courage, initiative, perseverance, skill, resource, and invention—in manual and other work for the war. We deal with the full list of 285 recipients of the Orders on page 443, so far as those honoured are workers in the fields covered by The Hospital. There remains the important consideration that sixty-four recipients of these Orders are women, and eleven other women have been awarded the new Empire Medal for services of special merit.

☛ Previous Articles appeared in The Hospital of August 4, 11, and 18.

In exact proportion to the knowledge of previous non-warrior Honours Lists possessed by the observer, will be the grateful appreciation arising from a close study of the names of the first recipients of the new Orders and the Medal for merit. That is praise indeed, but those who are responsible for the selections made have well earned it and deserve it. Of the 64 women who receive an Order, 5 have been made Dames Grand Cross (G.B.E.); 5 Dames Commanders (D.B.E.); 22 Commanders (C.B.E.); 14 Officers (O.B.E.); and 14 Members (M.B.E.).

All hospital workers especially, and every rank of His Majesty's subjects throughout the Empire, will be grateful to the King for his graceful acknowledgment of Her Majesty's devoted and ever-extending personal service in good works, especially since the commencement of the war, which her appointment to be a Dame Grand Cross of the British Empire fittingly recognises. Her Majesty the Queen is an untiring worker who never spares herself, and has, as her recent indisposition points, thought too little of her own health and too much, if that were possible, of the blessed claims and needs of a multitude of claimants for personal service on the part of the women of the nation, the number of whom have steadily increased as the war proceeds. Appreciation will be felt at the recognition of the cause of the Serbs by the honour paid to Lady Wemyss Paget for services to the Serbian Relief Fund. The home work of an army of British women receives recognition in the person of the Hon. Lady Lawley, who has been greatly instrumental in securing the success of Queen Mary's Needlework Guild as its honorary secretary. Mrs. Katherine Furse, the Commandant-in-Chief of the V.A.D. Corps, has proved herself to be far and away one of the very ablest women administrators whom we possess in this country to-day. She possesses the power of mastering the details of a great position, keeping herself in touch with everything going on in the wide field which she has to administer, and is invariably a cheerful, able, and encouraging personal factor in British war work by women. Lady Reid has been appointed a Dame Grand Cross of the British Empire for her splendid service in connection with the Australian Forces, a fact which will give gratification throughout the Empire.

A notable name is that of Mrs. Charles Lees, a former Mayor of Oldham, where she has done splendid personal service and genuine hard work with excellent results. Mrs. Charles Lees has been made a Dame Commander (D.B.E.). The nursing world will also welcome the bestowal of a Dame Commander on the Dowager Marchioness of Dufferin and Ava, C.I., whose Fund and work are known to many British nurses and hospital workers both at home and in India. The appointments of twenty-two Commanders of the British Empire (C.B.E.) include eight appointments given to women workers in hospitals and the nursing profession. Four are matrons of our oldest and greatest hospitals. They are in alphabetical order: Miss Margaret Hogg, of Guy's Hospital; Miss Eva Luckes, of the London Hospital; Miss Annie McIntosh; and Miss Lloyd Still, of St. Thomas's Hospital, and Superintendent of the Nightingale Training School for Nurses.

The self-denying life-work of Miss Luckes at the London Hospital, her exemplary devotion to duty, and her length of service (thirty-seven years) probably constitute a record. Miss Lloyd Still has shown great energy and much force and administrative ability at St. Thomas's Hospital and at the great training-school, over which she presides with such eminent success and general acceptance. Her work, too, on behalf of the College of Nursing will cause her to be held in grateful remembrance by the whole of the British nursing profession as time passes. It was a grateful act to confer a Commander of the British Empire on Sister Pauline, Sister of Charity of the Italian Hospital, London.

We cordially welcome, too, the appointment of Dr. Flora Murray to be a Commander of the British Empire. She has proved herself an able administrator who has done remarkable work as doctor in charge of Endell Street Hospital. Dr. Garrett Anderson, the organiser of the first hospital run by women at the Front, receives the same honour. We welcome, too, the honours conferred upon Lady Barrett and Dr. Mary Scharlieb, whose work is well known and deservedly appreciated throughout the medical profession.

It was our wish to attempt to do justice to the many Red Cross, V.A.D. hospital, social and charity workers who have been honoured by the King in the distribution of the new Orders and Medal. A fairly inclusive list of the names will be found on page 443, and we can only express our regret that absence of space prevents us from giving full details of the work done by every recipient of the new Orders and Medal.

We have pleasure in announcing that the French Government has conferred the Médaille d'Honneur d'Epidémies (argent) on Mrs. F. Bellville, matron of the Darrell Hospital, London, and the Hon. Katharine Norton, V.A.D., also of the Darrell Hospital, for their devotion to duty and good services rendered, whilst at No. 54 Hôpital Complémentaire, Dinard, France, in 1914-1915. The Crown Prince of Serbia has conferred on Mrs. Agnes Bennett, M.D., chairman of the English Hospital in Ostrovo, the Order of St. Sava, Class III. The brutal bombardment by the Germans of the Allies' hospitals in France has called forth an exhibition of bravery and courage on the part of all ranks of workers connected with them, including the doctors, matrons, sisters, nurses, and orderlies, worthy of the highest honour. The French Government have made Mdlle. de Baye, matron-in-charge of one of the bombed hospitals, a Chevalier of the Legion of Honour. They have also awarded the War Cross with Palms for distinguished services to four nurses

**ROUND THE HOSPITALS**—(*continued*).

working in the bombed hospitals for special bravery displayed during these bombardments. Mdlle. de Baye insisted on giving up her own steel helmet to put on a nurse whom she found working in a specially dangerous position.

There is an all but unanimous agreement amongst the authorities and matrons of British hospitals that a course of three years' training, under the best conditions, is a maximum course, when the sole aim is the training, and the knowledgeable and full equipment of a trained nurse of the highest standard. Our experience leads us to the conviction that, had it not been for the relatively constant, and always increasing, demands for more trained nurses, and the fact that the majority of such nurses have to be provided for and trained in the voluntary hospitals, the idea of a two years' certificate and of four years' service would never have arisen in Great Britain. The adoption of this latter plan rests for practical purposes on economic considerations, and it is certain that with the developments now taking place in the British nursing profession, including the founding of the College of Nursing, three years' training will become the British standard for training. Indeed, after the war we greatly doubt if any voluntary hospital will find it possible for any reasons, economic or otherwise, continuously to follow a course of training which involves four years' service in a hospital before a full certificate is issued by the authorities to the nurses trained within its walls.

We are led to these remarks by a study of the underlying causes of a controversy now proceeding in Australasia. The Federal Government of Australia called on Dr. Fetherston to report on the existing system of training nurses in Australasia, and this report was published on June 9, 1917. Dr. Fetherston condemns without qualification the four years' term, holding that a three years' course in general hospitals should be ample time for nurses to acquire a thorough practical training. Dr. Fetherston's view is supported by the fact that both the great nursing associations of Australasia—namely the Royal Victorian Trained Nurses' Association and the Australasian Trained Nurses' Association—accept a three years' course as sufficient. Dr. Fetherston, in speaking of the hospitals which enforce a four years' course, declares: "Under present conditions nurses in the public hospitals are subject, under the cloak of charity, to the grossest forms of sweating both as regards rates of pay and hours of work. The addition of an extra year to the three years' period for training merely carries the system of sweating still further."

It is not surprising that hospitals which have instituted a three years' term congratulate themselves and are glad. The Melbourne Hospital, with a four years' course, we are informed, has refused to alter its system, despite Dr. Fetherston's report, but the Adelaide General Hospital is reported to have set a patriotic example by modifying its system so as to allow the probationers attached to its school, at the end of two and a half years training, to present themselves for the final examination, and, when successful, has permitted them to be sisters for the remaining six months of their course at an increased salary equivalent to £50 per annum. This concession has enabled some units of the trained staff working at the Adelaide General Hospital to be released for war service. Dr. Fetherstoh estimates that about 500 nurses in Australia each year obtain their certificates and become fully trained. At the May examination this year of the Royal Victorian Trained Nurses' Association only seventy-seven nurses from twenty-two hospitals passed in Victoria, including twenty-one from the Melbourne Hospital. Of the seventy-seven nurses only two "took credit," both being from the Children's Hospital, and trained there under the old three years' term. Their names are Eleanor Archer and Dorothy Stephens. A proposal made by the Royal Victorian Trained Nurses' Association to increase the term of training for nurses from three to five years has been abandoned.

The supply of promising and suitable probationers for training is likely to become a pressing question. The circumstances of the moment, offering as they do many fields of work to women, reveal with increasing force the fact that the highest type of recruit does not offer, as was once the case. The matron of a provincial hospital gives it as her experience that the unselfish devotion of the best nurses can scarcely be hoped for. Miss Nightingale's call roused women not afraid to face weariness and painfulness. In their enthusiasm long hours and few holidays were not counted as grievous, for they were women who gloried in adversity. Our matron correspondent feels that the College of Nursing may, by making the profession like one of the Services now fighting for us, raise again the standard, which may otherwise grow less noble. Such a development is needed, she declares, at least for the smaller institutions. Her experience is, and we incline to agree with her, that a hospital with a medical school attached is less likely to deteriorate. On the other hand, embryo probationers and their friends should thank God and take courage that they live in the present times, for after the war, the spirit of the whole Nursing Service will be quickened, and the glory of being a highly trained and competent nurse will be greater than ever. The truth is, the war has brought the importance of a trained nurse's services home to all classes of people everywhere. Consequently, her status, emoluments, and position, when she is faithful, should be, after the war, higher and better than ever before in the history of the world.

☛ It is our invariable practice to pay for all items of news which we may publish. The minimum payment is 5s. Paragraphs of about twenty lines in length—that is to say, of 150 words or less—are preferred. Contributions should be addressed to The Editor, The Hospital, 28 and 29 Southampton Street, Strand, London, W.C., and should reach him by the first post on Mondays.

